# Supplementary material for: pH-Triggered Hydrogel Nanoparticles for Efficient Anticancer Drug Delivery and Bioimaging Applications
Source: Pharmaceutics. 2024 Jul 11;16(7):931. doi: 10.3390/pharmaceutics16070931 (PMC11279682; doi:10.3390/pharmaceutics16070931)
Supplement: Supplementary file 1 [file pharmaceutics-16-00931-s001.zip › pharmaceutics-3088518-supplementary.pdf]

pharmaceutics

*Supplementary information*

# pH-Triggered Hydrogel Nanoparticles for Efficient Anticancer Drug Delivery and Bioimaging Applications

**Keristina Wagdi K. Amin [1,2], Ágota Deák [1], Miklós Csanády, Jr. [3], Nikoletta Szemerédi [4], Diána Szabó [3], Árpád Turcsányi [5], Ditta Ungor [5], Gabriella Spengler [4], László Rovó [3] and László Janovák [1,*]**

1 Department of Physical Chemistry and Materials Science, University of Szeged, Rerrich Béla tér 1, H-6720 Szeged, Hungary; keristina.wagdi@chem.u-szeged.hu (K.W.K.A.); agotadeak@chem.u-szeged.hu (Á.D.)
2 Department of Chemistry, Faculty of Science, Suez Canal University, Ismailia 41522, Egypt
3 Department of Oto-Rhino-Laryngology and Head & Neck Surgery, University of Szeged, Tisza Lajos krt. 111, H-6724 Szeged, Hungary; csanady.miklos.2@med.u-szeged.hu (M.C.J.); diniklinik@freemail.hu (D.S.); office.orl@med.u-szeged.hu (L.R.)
4 Department of Medical Microbiology, Albert Szent-Györgyi Medical School, University of Szeged, H-6725 Szeged, Hungary; szemeredi.nikoletta@med.u-szeged.hu (N.S.); spengler.gabriella@med.u-szeged.hu (G.S.)
5 MTA-SZTE Lendület "Momentum" Noble Metal Nanostructures Research Group, University of Szeged, Rerrich B. sqr. 1, H-6720 Szeged, Hungary; tarpad@chem.u-szeged.hu (Á.T.); ungord@chem.u-szeged.hu (D.U.)
* Correspondence: janovakl@chem.u-szeged.hu; Tel.: +36-62-544-210; Fax: +36-62-544-042

## Materials and Methods

*Materials needed for the synthesis of succinic anhydride precursor and PVA-SA*

Succinic acid ($C_4H_6O_4$) was obtained from Reanal, Hungary. Polyvinyl alcohol (PVA, 86–89% degree of hydrolysis, Mw 46.8 kDa) was purchased from Nagart Kft., Hungary. Sodium acetate ($C_2H_3NaO_2$), and acetic anhydride ($C_4H_6O_3$) were obtained from Molar Chemicals Kft., Hungary. Diethyl ether was acquired from VWR Chemicals BDH®, while *N,N*-Dimethylformamide (DMF) was acquired from Merck, Germany.

*Synthetic procedures of succinic anhydride precursor and PVA-SA*

In order to synthesize PVA-SA, it was necessary to first prepare succinic anhydride, as mentioned in our previous study [8]. In brief, 25 mL of acetic anhydride were added to 15 g of succinic acid under a nitrogen atmosphere. This mixture was placed in a round-bottom flask, which was fitted with a reflux condenser and an anhydrous calcium chloride drying tube. Next, a steam bath was used to gently heat the reaction until it reached a clear solution. After that, the reaction was heated for one hour to ensure completion. Then, the white succinic anhydride crystals were produced by cooling down the mixture in an ice bath and obtained by filtration. To wash these crystals, a small quantity of diethyl ether was utilized, followed by drying under vacuum. These synthetic procedures end up with almost 10.5 g of purified crystals. Next, the prepared succinic anhydride was used to modify PVA, as was described earlier [8]. Briefly, 9 mL of DMF was used to dissolve 0.5 g of succinic anhydride and 1 g of PVA. Then, anhydrous sodium acetate (0.05 g) was introduced to speed up the reaction. The reaction was then stirred continuously at 40 °C for one day. After that, the reaction product was acquired through the addition of diethyl ether. This crude product was rinsed several times with ether and ethanol, and then the washed product was allowed to dry under vacuum.

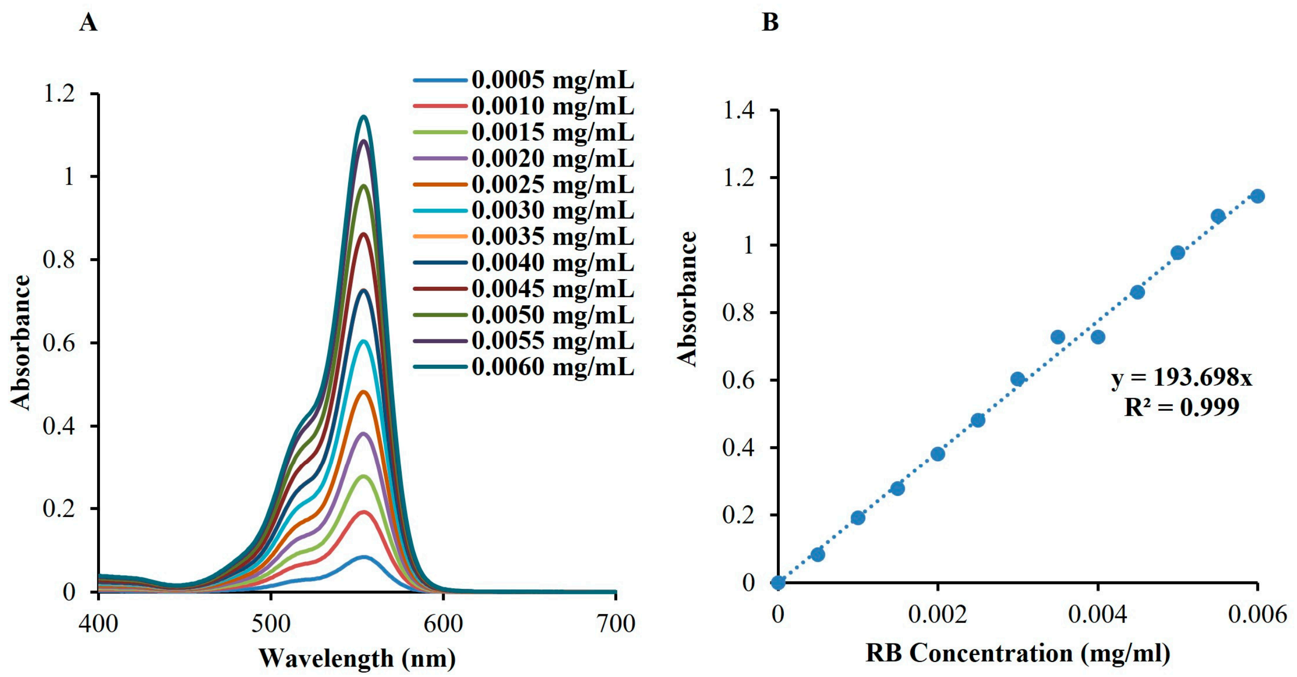


**Figure S1.** UV-Vis absorption spectra (**A**) and calibration plot (**B**) for RB in MQ water.

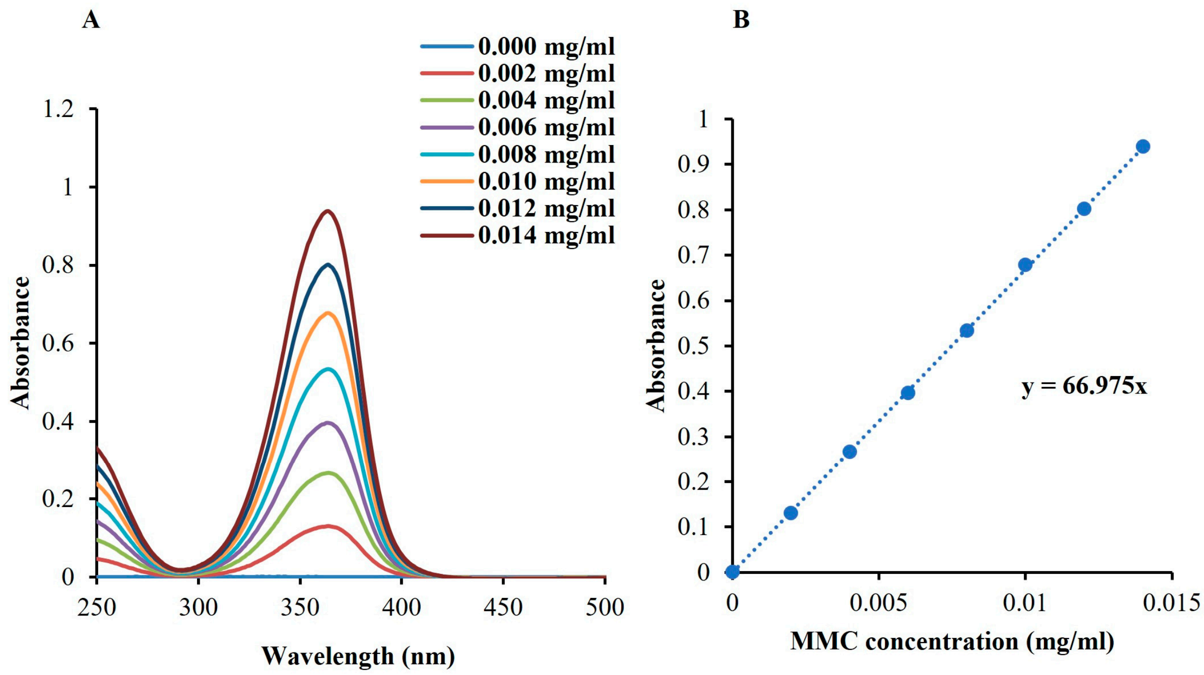


**Figure S2.** UV-Vis absorption spectra (**A**) and calibration plot (**B**) for MMC in MQ water.

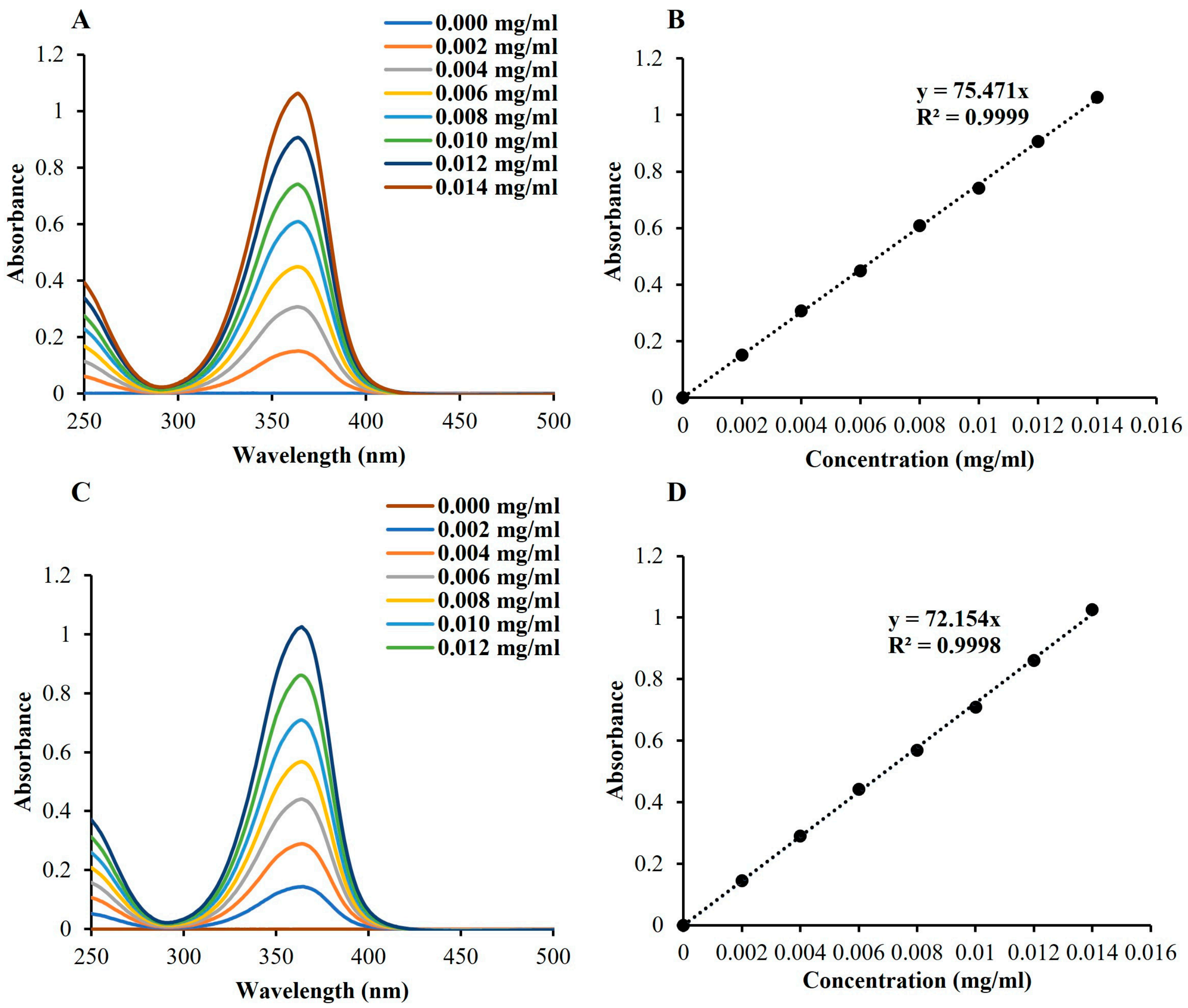


**Figure S3.** UV-Vis absorption spectra and calibration plot for MMC in phosphate buffered saline solution (PBS) with a pH of 7.4 (**A** and **B**) and 5.8 (**C** and **D**).

pharmaceutics

MDPI

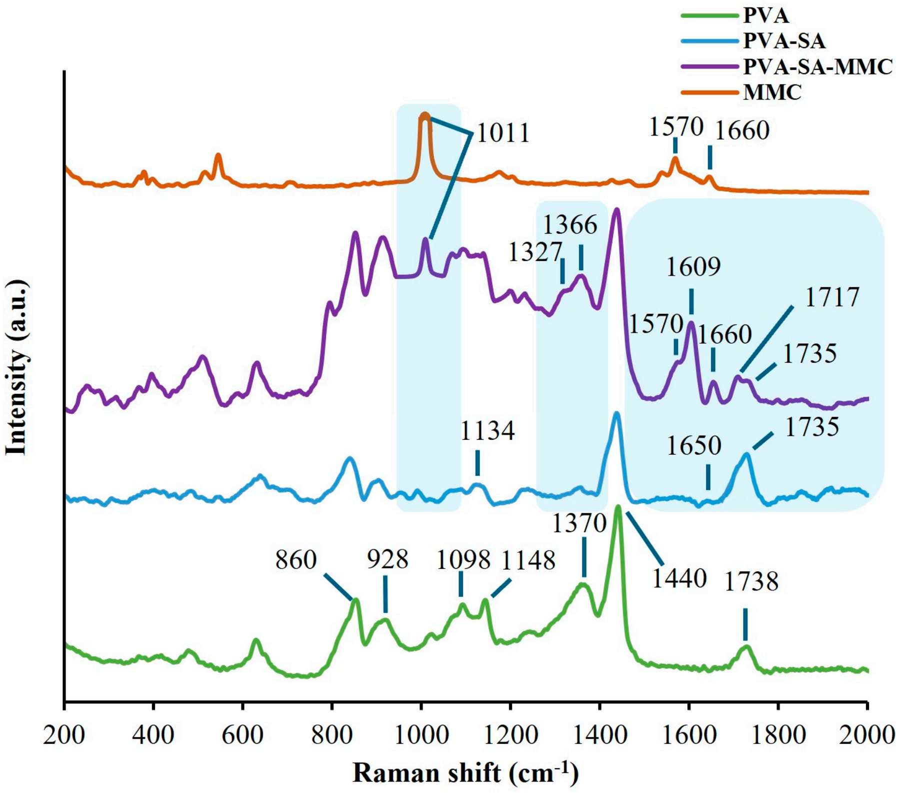


**Figure S4**. Raman spectra of PVA, PVA-SA, PVA-SA-MMC and MMC.

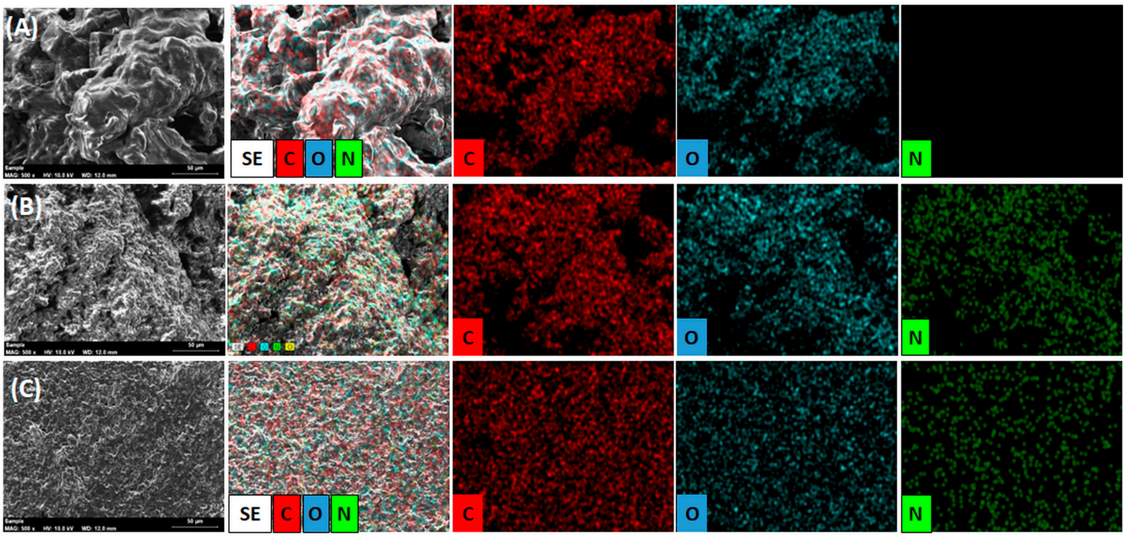


**Figure S5**. EDX of (**A**) PVA-SA and (**B**) RB-PVA-SA as well as (**C**) RB-PVA-SA-MMC, where C,O, and N are carbon, oxygen, and nitrogen elements, respectively.

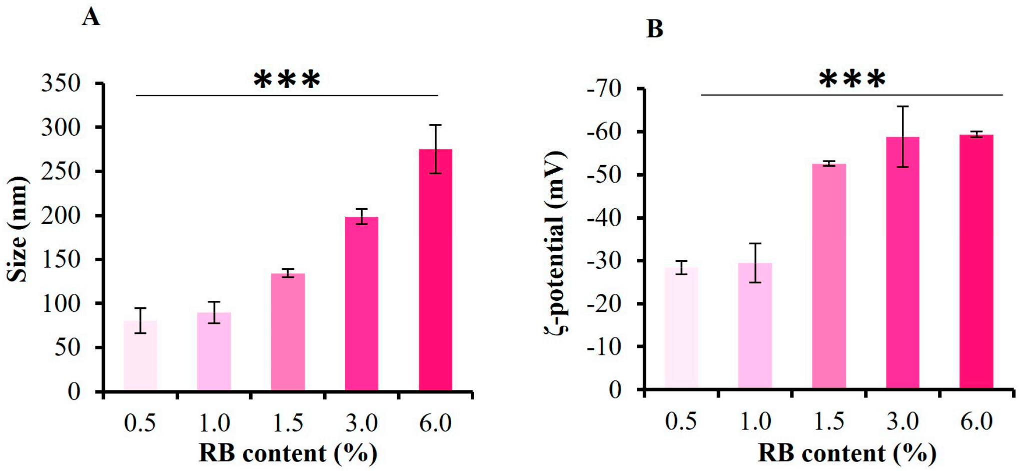


**Figure S6.** The average particle sizes (**A**) and the ζ potential values (**B**) of the produced RB-loaded NPs at pH 5.8. Statistical analysis of the results: the added asterisks in the figure represent the summarized p value obtained from one-way ANOVA analysis: * $p<0.05$; ** $p<0.01$; *** $p<0.001$.

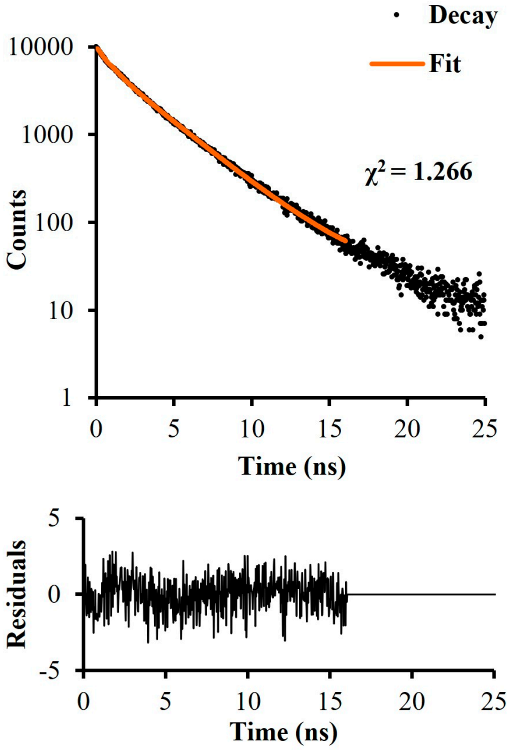


**Figure S7.** The typical fluorescence decay profile for (1.5% RB)-PVA-SA NPs with the fitting.

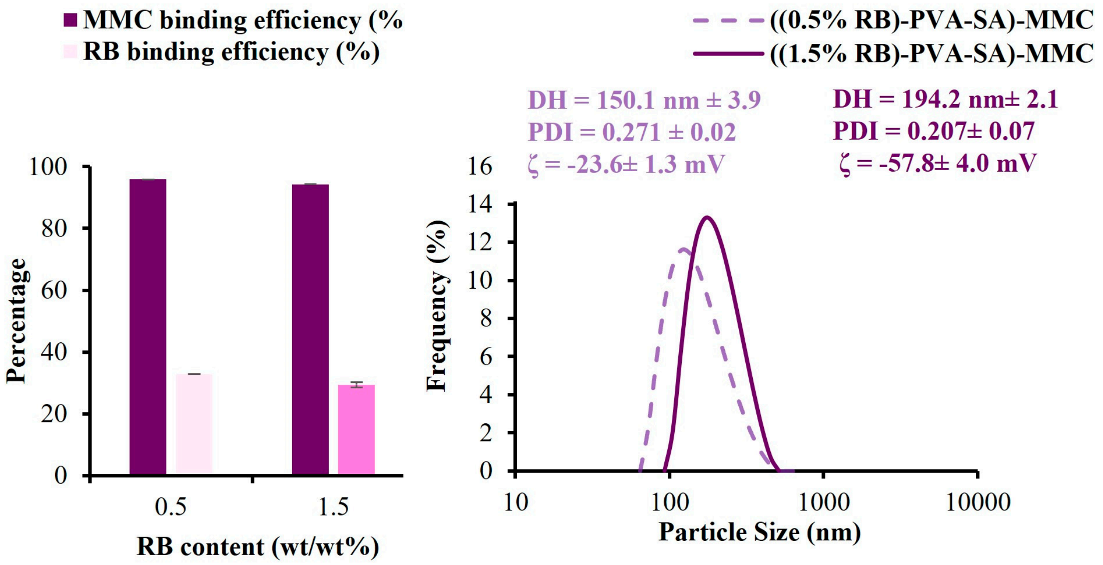


**Figure S8.** The MMC and RB binding efficiency results (**A**) as well as the DLS data at pH 7.4 (**B**) for the two samples obtained from applying the second MMC concentration (1.2 mg/ml) either with a 0.5% or 1.5% RB.

**Table S1**. Results of Tukey's multiple comparisons test on dye loading percentage, particle diameters, and ζ-potential values of the produced RB-loaded NPs.

| | Tukey's multiple comparisons | P Value | Significant? | Summary |
|---|---|---|---|---|
| **Dye loading results (presented in Figure 4A)** | 0.5% RB vs. 1.0 % RB | 0.65 | No | ns |
| | 0.5% RB vs. 1.5 % RB | 0.02 | Yes | * |
| | 0.5% RB vs. 3.0 % RB | <0.001 | Yes | *** |
| | 0.5% RB vs. 6.0 % RB | <0.001 | Yes | *** |
| | 1.0 % RB vs. 1.5 % RB | 0.19 | No | ns |
| | 1.0 % RB vs. 3.0 % RB | <0.001 | Yes | *** |
| | 1.0 % RB vs. 6.0 % RB | <0.001 | Yes | *** |
| | 1.5 % RB vs. 3.0 % RB | <0.001 | Yes | *** |
| | 1.5 % RB vs. 6.0 % RB | <0.001 | Yes | *** |
| | 3.0 % RB vs. 6.0 % RB | <0.001 | Yes | *** |
| **Particle diameter results (presented in Figure 4B)** | 0.5% RB vs. 1.0 % RB | 0.62 | No | ns |
| | 0.5% RB vs. 1.5 % RB | <0.001 | Yes | *** |
| | 0.5% RB vs. 3.0 % RB | <0.001 | Yes | *** |
| | 0.5% RB vs. 6.0 % RB | <0.001 | Yes | *** |
| | 1.0 % RB vs. 1.5 % RB | 0.002 | Yes | ** |
| | 1.0 % RB vs. 3.0 % RB | <0.001 | Yes | *** |
| | 1.0 % RB vs. 6.0 % RB | <0.001 | Yes | *** |
| | 1.5 % RB vs. 3.0 % RB | <0.001 | Yes | *** |
| | 1.5 % RB vs. 6.0 % RB | <0.001 | Yes | *** |
| | 3.0 % RB vs. 6.0 % RB | <0.001 | Yes | *** |
| **ζ-potential results (presented in Figure 4D)** | 0.5% RB vs. 1.0 % RB | 0.94 | No | ns |
| | 0.5% RB vs. 1.5 % RB | <0.001 | Yes | *** |
| | 0.5% RB vs. 3.0 % RB | <0.001 | Yes | *** |
| | 0.5% RB vs. 6.0 % RB | <0.001 | Yes | *** |
| | 1.0 % RB vs. 1.5 % RB | <0.001 | Yes | *** |
| | 1.0 % RB vs. 3.0 % RB | <0.001 | Yes | *** |
| | 1.0 % RB vs. 6.0 % RB | <0.001 | Yes | *** |
| | 1.5 % RB vs. 3.0 % RB | 0.14 | No | ns |
| | 1.5 % RB vs. 6.0 % RB | 0.11 | No | ns |
| | 3.0 % RB vs. 6.0 % RB | >0.99 | No | ns |

**Table S2**. The DLS data (at pH 5.8) for the two samples obtained from applying the first MMC concentration (0.4 mg/ml) either with a 0.5% or 1.5% RB.

| Sample | DH (nm) | PDI | ζ-potential (mV) |
|---|---|---|---|
| ((0.5% RB)-PVA-SA)-MMC | 127 ± 3 | 0.276 ± 0.02 | -27.9 ± 0.7 |
| ((1.5% RB)-PVA-SA)-MMC | 156 ± 23 | 0.214 ± 0.07 | -54.8 ± 2.0 |

**Table S3**. The outcomes of fitting the release data to various kinetic models.

| | *Zero-Order model* | | *First-Order model* | | *Higuchi model* | | *Hixson-Crowell model* | | *Korsmeyer-Peppas model* | | |
|---|---|---|---|---|---|---|---|---|---|---|---|
| | $R^2$ | $k(h^{-1})$ | $R^2$ | $k(h^{-1})$ | $R^2$ | $k(h^{-1/2})$ | $R^2$ | $k(h^{-1/3})$ | $R^2$ | $n$ | $k(h^{-n})$ |
| **Free MMC (pH 7.4)** | 0.312 | 0.164 | 0.426 | 0.007 | 0.499 | 2.996 | 0.390 | 0.006 | 0.702 | 0.148 | 48.31 |
| **Free MMC (pH 5.8)** | 0.106 | 0.140 | 0.149 | 0.005 | 0.224 | 2.891 | 0.134 | 0.005 | 0.537 | 0.200 | 49.43 |
| **((0.5% RB)-PVA-SA)-MMC (pH 7.4)** | 0.966 | 0.221 | 0.987 | 0.002 | 0.986 | 3.637 | 0.981 | 0.004 | 0.988 | 0.771 | 0.818 |
| **((0.5% RB)-PVA-SA)-MMC (pH 5.8)** | 0.960 | 0.409 | 0.983 | 0.009 | 0.965 | 6.084 | 0.985 | 0.010 | 0.988 | 0.771 | 1.403 |
| **((1.5% RB)-PVA-SA)-MMC (pH 7.4)** | 0.970 | 0.101 | 0.978 | 0.001 | 0.971 | 1.418 | 0.976 | 0.002 | 0.979 | 0.625 | 0.701 |
| **((1.5% RB)-PVA-SA)-MMC (pH 5.8)** | 0.950 | 0.325 | 0.988 | 0.005 | 0.975 | 4.763 | 0.980 | 0.007 | 0.991 | 0.831 | 0.861 |
